# Supplementary material for: Phosphoproteomic identification of ULK substrates reveals VPS15‐dependent ULK/VPS34 interplay in the regulation of autophagy
Source: EMBO J. 2021 Jun 14;40(14):e105985. doi: 10.15252/embj.2020105985 (PMC8280838; doi:10.15252/embj.2020105985)
Supplement: Supplementary file 1 — Appendix [file EMBJ-40-e105985-s007.docx]

Appendix: ULK substrate screens provide novel roles for ULK and VPS34 complexes in the regulation of autophagy

Table of Contents:

Supplementary Tables (S1-S3)

**Table S1.** *Putative substrate shortlist.*

Potential ULK substrates are given, with screen identified, gene names and both human and murine residue numbers included. Putative phosphoacceptors with ≥5 mismatches within 15mer between human and murine homologs were excluded. Where multiple sites in close proximity with similar profiles were identified, all were included with residues separated by a stroke. Peptides satisfying all 3 TMT variables (see Fig EV1E) are indicated in the fifth column - asterisks represent phosphopeptides matching 2 variables only that fulfilled additional criteria (or, in the case of ANXA2, had an established role in autophagy) and were thus included. Phosphopeptides with profiles similar to Prkab2 S38 (Pearson correlation coefficient >0.7) are indicated in the sixth column, and the seventh column shows phosphopeptides with an average log_2_(DKO/WT) value of <-1 SILAC experiment 1.

| **Screen** | **Gene Name** | **Human Residue(s)** | **Murine Residue(s)** | **Profile Match** | **Prkab2 CorCoeff** | **SILAC Experiment** |
| --- | --- | --- | --- | --- | --- | --- |
| TMT | ACTG1 | S33 | S33 | y | n | - |
|  | ANXA2 | S127 | S127 | y* | n | n |
|  | CHEK1 | S317 | S317 | y | y | - |
|  | CTNND1 | S920 | S819 | y | n | n |
|  | LAP3 | S238 | S238 | y | y | n |
|  | MSL3 | S315 | S311 | y | n | - |
|  | NEDD4L | T302 | T182 | y | y | - |
|  | PCM1 | S110 | S110 | y | n | n |
|  | PRKAB2 | S44 | S43 | y | y | - |
|  | RALGPS2 | S289/T290 | S289/T290 | y* | y | - |
|  | SCEL | S91 | S90 | y | y | - |
|  | SHROOM1 | S49 | S49 | y | n | - |
|  | STAG2 | S1198 | S1198 | y | n | n |
|  | TBC1D1 | S627 | S621 | y | n | n |
|  | VIM | T426 | T426 | y | n | n |
|  | VPS26B | S302/S304 | S302/S304 | y | n | n |
|  | ZNF800 | S462 | S460 | y | y | - |
| TMT + SILAC | CARS | S307 | S390 | y | y | y |
|  | F11R | S287 | S288 | y* | y | y |
|  | GPRC5A | S301 | S303 | y | y | y |
|  | NHSL1 | S190 | S189 | y* | n | y |
|  | SHROOM2 | S231 | S236 | y | y | y |
|  | SORBS2 | S298/S299/S301/S302 | S442/S443/S445/S446 | y | y | y |
|  | VAMP5 | S41 | S22 | y | y | y |
|  | VILL | S233 | S234 | y* | y | y |
| SILAC | PRKAG2 | S122 | S122 | - | - | y |
|  | SORBS2 | S239 | S383 | - | - | y |
|  | VPS15 | S861 | S861 | - | - | y |

**Table S2.** *Peptide array-based in vitro kinase assay sequences and results.*

Amino acid sequences of peptides synthesised for the *in vitro kinase* assay described in Fig EV2. Positive controls are listed first followed by putative substrates grouped according to the screens in which they were identified. The ‘Name’ column lists human gene name followed by phosphoacceptor residue and number, along with encoded mutation. For total phosphomutant peptides (S/T-AA), all serine and threonine residues were changed to alanine. ‘ULKtide_S’ refers to the optimal ULK1 substrate peptide [24], with ‘ULKtide_A’ encoding the serine to alanine (i.e. non-phosphorylatable) form. The mutations introduced to ‘murinise’ peptides are described in the ‘Name’ column where appropriate. The putative phosphoacceptor residue is shown in bold in the ‘Sequence’ column. The final column indicates phosphorylation status of peptides in assay. Relative phosphorylation level was measured by densitometry. WT, single phosphomutant or murinised peptides that were phosphorylated more efficiently than the total phosphomutant peptide are marked with a ‘y’. WT, single phosphomutant or murinised peptides that were not phosphorylated more than the total phosphomutant are marked with a ‘n’. All total phosphomutant peptides were considered unphosphorylated and marked with an ‘n’. Of note, ‘murinised’ VILL S233 was excluded from analysis due to a technical error in peptide array generation.

| Name | Sequence | Phosphorylated |
| --- | --- | --- |
| ATG13_S355 | EDTETVSN**S**SEGRAS | n |
| ATG13_S355A | EDTETVSN**A**SEGRAS | n |
| ATG14_S29 | PLARDLVD**S**VDDAEG | y |
| ATG14_S29A | PLARDLVD**A**VDDAEG | n |
| BECN1_S15 | SNNSTMQV**S**FVCQRC | y |
| BECN1_S15A | SNNSTMQV**A**FVCQRC | n |
| NR3C2_S843 | FNEEKMHQ**S**AMYELC | y |
| NR3C2_S843A | FNEEKMHQ**A**AMYELC | n |
| PRKAB2_S39 | HKIMVG**S**TDDPSVFSLPDS | y |
| PRKAB2_S39A | HKIMVG**A**TDDPSVFSLPDS | y |
| ULK1_S1042_T1046 | CIERRL**S**ALL**T**GICA | n |
| ULK1_S1042A_T1046A | CIERRL**A**ALL**A**GICA | n |
| ULKtide_S | YANWLAA**S**IYLDGKKK | y |
| ULKtide_A | YANWLAA**A**IYLDGKKK | n |

**Positive Controls**

**TMT**

| Name | Sequence | Phosphorylated |
| --- | --- | --- |
| ACTG1_S33 | DAPRAVFP**S**IVGRPR | y |
| ACTG1_S33A | DAPRAVFP**A**IVGRPR | n |
| ANXA2_S127 | KGLGTDED**S**LIEIIC | y |
| ANXA2_S127A | KGLGTDED**A**LIEIIC | n |
| ANXA2_S/T-AA | KGLGADED**A**LIEIIC | n |
| CHEK1_S317 | EENVKYSS**S**QPEPRT | y |
| CHEK1_S317A | EENVKYSS**A**QPEPRT | y |
| CHEK1_S/T-AA | EENVKYAA**A**QPEPRA | n |
| CHEK1_S317_N311T/Y314F | EETVKFSS**S**QPEPRT | y |
| CTNND1_S920 | DHNRTLDR**S**GDLGDM | y |
| CTNND1_S920A | DHNRTLDR**A**GDLGDM | n |
| CTNND1_S/T-AA | DHNRALDR**A**GDLGDM | n |
| LAP3_S238 | TEVHIRPK**S**WIEEQA | y |
| LAP3_S238A | TEVHIRPK**A**WIEEQA | n |
| LAP3_S/T-AA | AEVHIRPK**A**WIEEQA | n |
| MSL3_S315 | RSQEELSP**S**PPLLNP | n |
| MSL3_S315A | RSQEELSP**A**PPLLNP | n |
| MSL3_S/T-AA | RAQEELAP**A**PPLLNP | n |
| NEDD4L_T302 | PPASPGSR**T**SPQELS | n |
| NEDD4L_T302A | PPASPGSR**A**SPQELS | n |
| NEDD4L_S/T-AA | PPAAPGAR**A**APQELA | n |
| NEDD4L_T302_G299V | PPASPVSR**T**SPQELS | n |
| PCM1_S110 | KLKQRINF**S**DLDQRS | y |
| PCM1_S110A | KLKQRINF**A**DLDQRS | n |
| PCM1_S/T-AA | KLKQRINF**A**DLDQRA | n |
| PRKAB2_S44 | HKIMVGSTDDP**S**VFSLPDS | y |
| PRKAB2_S44A | HKIMVGATDDP**S**VFSLPDS | y |
| PRKAB2_S/T-AA | HKIMVGAADDP**A**VFALPDA | n |
| RALGPS2_S289/T290 | SLKIEPG**TS**TPRSAA | y |
| RALGPS2_S289/T290A | SLKIEPG**AA**TPRSAA | y |
| RALGPS2_S/T-AA | ALKIEPG**AA**APRAAA | n |
| SCEL_S91 | KATISRYS**S**DDTLDR | y |
| SCEL_S91A | KATISRYS**A**DDTLDR | y |
| SCEL_S/T-AA | KAAIARYA**A**DDALDR | n |
| SCEL_S91_S90R/D92E/T93M | KATISRYR**S**EDMLDR | y |
| SHROOM1_S49 | GGPEPRTQ**S**PGTDLL | n |
| SHROOM1_S49A | GGPEPRTQ**A**PGTDLL | n |
| SHROOM1_S/T-AA | GGPEPRAQ**A**PGADLL | n |
| SHROOM1_S49_Q48P | GGPEPRTP**S**PGTDLL | n |
| STAG2_S1198 | QHAIRRGT**S**LMEDDE | n |
| STAG2_S1198A | QHAIRRGT**A**LMEDDE | n |
| STAG2_S/T-AA | QHAIRRGA**A**LMEDDE | n |
| TBC1D1_S627 | QRKLMRYH**S**VSTETP | y |
| TBC1D1_S627A | QRKLMRYH**A**VSTETP | n |
| TBC1D1_S/T-AA | QRKLMRYH**A**VAAEAP | n |
| VIM_T426 | FSSLNLRE**T**NLDSLP | y |
| VIM_T426A | FSSLNLRE**A**NLDSLP | y |
| VIM_S/T-AA | FAALNLRE**A**NLDALP | n |
| VPS26B_S302/304 | RKGDIVRK**S**M**S**HQAA | y |
| VPS26B_S302/304A | RKGDIVRK**A**M**A**HQAA | n |
| ZNF800_S462 | DSESPKST**S**PSAAGG | n |
| ZNF800_S462A | DSESPKST**A**PSAAGG | n |
| ZNF800_S/T-AA | DAEAPKAA**A**PAAAGG | n |

**TMT + SILAC**

| Name | Sequence | Phosphorylated |
| --- | --- | --- |
| CARS_S307 | EGEGDLSI**S**ADRLSE | y |
| CARS_S307A | EGEGDLSI**A**ADRLSE | n |
| CARS_S/T-AA | EGEGDLAI**A**ADRLAE | n |
| F11R_S287 | IYSQPSAR**S**EGEFKQ | y |
| F11R_S287A | IYSQPSAR**A**EGEFKQ | n |
| F11R_S/T-AA | IYAQPAAR**A**EGEFKQ | n |
| F11R_S287_A285T | IYSQPSTR**S**EGEFKQ | y |
| GPRC5A_S301 | YGVENRAY**S**QEEITQ | n |
| GPRC5A_S301A | YGVENRAY**A**QEEITQ | n |
| GPRC5A_S/T-AA | YGVENRAY**A**QEEIAQ | n |
| NHSL1_S190 | DRQASLRR**S**LIYTDT | y |
| NHSL1_S190A | DRQASLRR**A**LIYTDT | y |
| NHSL1_S/T-AA | DRQAALRR**A**LIYADA | n |
| SHROOM2_S231 | TLSKADTS**S**AENILY | n |
| SHROOM2_S231A | TLSKADTS**A**AENILY | n |
| SHROOM2_S/T-AA | ALAKADAA**A**AENILY | n |
| SHROOM2_S231_S225P/T229A/A232T | TLPKADAS**S**TENILY | n |
| SORBS2_S298/299/301/302 | LTKSFTS**SS**P**SS**PSRAKGG | y |
| SORBS2_ S298/299/301/302A | LTKSFTS**AA**P**AA**PSRAKGG | y |
| SORBS2_S298/299/301/302A_S/T-AA | LAKAFAA**AA**P**AA**PARAKGG | n |
| SORBS2_S298/299/301/302_T296I/L291F | FTKSFIS**SS**P**SS**PSRAKGG | y |
| VAMP5_S41 | KLAELQQR**S**DQLLDM | n |
| VAMP5_S41A | KLAELQQR**A**DQLLDM | n |
| VAMP5_S41_Q38E | KLAELEQR**S**DQLLDM | n |
| VILL_S233 | AVLGRRVG**S**LRAATP | y |
| VILL_S233A | AVLGRRVG**A**LRAATP | n |
| VILL_S/T-AA | AVLGRRVG**A**LRAAAP | n |

**SILAC**

| Name | Sequence | Phosphorylated |
| --- | --- | --- |
| PRKAG2_S122 | PPRSPRRM**S**FSGIFR | y |
| PRKAG2_S122A | PPRSPRRM**A**FSGIFR | y |
| PRKAG2_S/T-AA | PPRAPRRM**A**FAGIFR | n |
| SORBS2_S239 | LYQSSIDR**S**LERPMS | y |
| SORBS2_S239A | LYQSSIDR**A**LERPMS | y |
| SORBS2_S239A_S/T-AA | LYQAAIDR**A**LERPMA | n |
| VPS15_S861 | SNVNEEWK**S**MFGSLD | y |
| VPS15_S861A | SNVNEEWK**A**MFGSLD | n |

**Table S3.** *Summary of the analysis for the quantification of WIPI2 puncta by high throughput screening in Fig EV5A.*

| Step | Building Block | Input / Method | Output |
| --- | --- | --- | --- |
| 1 | Input image | Flatfield Correction: Basic / Stack Processing: Maximum Projection / Min. Global Binning: Dynamic |  |
| 2 | Calculate Image | Method: Formula, Formula: A+B, Channel A: Alexa 488, Channel B: Alexa 555 | 488 + 555 mask |
| 3 | Find Nuclei | Channel: HOECHST 33342 / Method: B, Common Threshold: 0.3, Area: 40µm^2^, Splitting Coefficient: 9.0, Individual Threshold: 0.35, Contrast: >0.1 | Nuclei |
| 4 | Calculate Morphology Properties | Population: Nuclei / Method: Standard, Area |  |
| 5 | Select Population | Population: Nuclei / Method: Filter by Property, Nucleus area [px2]: > 900 | Nuclei Selected |
| 6 | Find Cytoplasm | Channel: 488 + 555 mask, Nuclei: Nuclei Selected / Method: A, Individual Threshold: 0.25 |  |
| 7 | Calculate Intensity Properties | Channel: Alexa 488, Population: Nuclei Selected, Region: Cell / Method: Standard, Mean | Intensity Cell Alexa 488 |
| 8 | Select Population | Population: Nuclei Selected / Method: Filter by Property, Intensity Cell Alexa 488 Mean: > 235 | 488 Pos Cell Total |
| 9 | Find Spots | Channel: Alexa 555, ROI: Nuclei Selected, ROI Region: Cell / Method: C, Radius: <= 2.2 px, Contrast: > 0.11, Uncorrected Spot to Region Intensity: > 1.3, Distance: >= 3 px, Spot Peak Radius: 0 px, Calculate Spot Properties | Spots |
| 10 | Calculate Intensity Properties | Channel: Alexa 555, Population: Spots, Region: Spot / Method: Standard, Mean | Intensity Spot Alexa 555 |
| 11 | Select Population | Population: Spots / Method: Filter by Property, Corrected Spot Intensity: > 12, Spot to Region Intensity: > 1.5, Boolean Operation: F1 OR F2 | WIPI Pos Puncta |
| 12 | Calculate Properties | Population: Nuclei Selected / Method: By Related Population, Related Population: WIPI2 Pos Puncta, Number of WIPI2 Pos Puncta | Property Suffix: per Cell |
| 13 | Select Population | Population: Nuclei Selected / Method: Filter by Property, Number of WIPI2 Pos Puncta- per cell: >= 1 | WIPI2 Pos Cell |
| 14 | Calculate Properties | Population: 488 Pos Cell Total / Method: By Related Population, Related Population: WIPI2 Pos Puncta, Number of WIPI2 Pos Puncta | Property Suffix: per Cell |
| 15 | Define Result | Object Results: Nuclei Selected, WIPI2 Pos Puncta, Number of WIPI2 Pos Puncta- per Cell |  |
