# Supplementary material for: Phosphoproteomic identification of ULK substrates reveals VPS15‐dependent ULK/VPS34 interplay in the regulation of autophagy
Source: EMBO J. 2021 Jun 14;40(14):e105985. doi: 10.15252/embj.2020105985 (PMC8280838; doi:10.15252/embj.2020105985)
Supplement: Supplementary file 2 — Expanded View Figures PDF [file EMBJ-40-e105985-s008.pdf]

Expanded View Figures

Figure EV1. Identification of novel ULK substrates via phosphoproteomic analyses.

A Heat map with SILAC ratios for phosphopeptides with an average $\log_2$[DKO/WT] value of < -1.5 in forward (fwd) and reverse (rev) labelling permutations from at least 1 of 3 independent experiments displayed, excluding those showing label-dependent enrichment in any of the 3 control conditions ($-0.5<\log_2$[WT/WT]>0.5). Colour intensity indicates $\log_2$ SILAC enrichment, as indicated in colour key.

B Curated human protein–protein interaction network based on interactions from Bioplex (Huttlin *et al*, 2017), CORUM, (Giurgiu *et al*, 2019) and Autophagy Regulatory Network databases (Türei *et al*, 2015), depicting selected proteins within 2 nodes of ULK1 or ULK2 (bold). Proteins containing phosphopeptides depleted in DKOs (see Table EV1) are annotated in red. GABARAP was identified in a previously published screen for autophagy regulators (McKnight *et al*, 2012).

C Correlation cluster analysis on the 2,926 most variable phosphopeptide profiles revealed 6 basic patterns. For each group, the number of constituent profiles is given with WT enrichment values plotted in green and DKO in red. Thick green and red lines reveal the average WT and DKO profiles, respectively, with thin translucent lines revealing individual profiles. Log_{10}(TMT Enrichment) values for each phosphopeptide were normalised to DKO 0 min with relative enrichment plotted on the Y axis.

D Phosphopeptides reproducibly depleted in the SILAC analysis (Table EV1) were cross-referenced with the TMT data set, with profiles (100 total) plotted as in C.

E TMT enrichment values for the above described SILAC sites were displayed in a heat map. Columns are populated by the 10 time course conditions and rows by specific phosphopeptides. Cell colour represents relative enrichment, calculated by averaging values across all 10 conditions, dividing the enrichment value at each time point by this value, then plotting the logarithm of this fraction to base 2 (see colour bar). The 6 most common groups after global correlation cluster analysis are annotated on the left. The red cluster is largely populated with phosphopeptides displaying little variation between cell types and time course conditions.

F Phosphorylation profiles for Prkab2 S38 plotted as in Fig 1E. The variables used to triage the TMT data set are depicted as arrows with colour coded descriptions of thresholds used below (units for thresholds = $\log_{10}$(TMT Enrichment)).

© 2021 The Authors

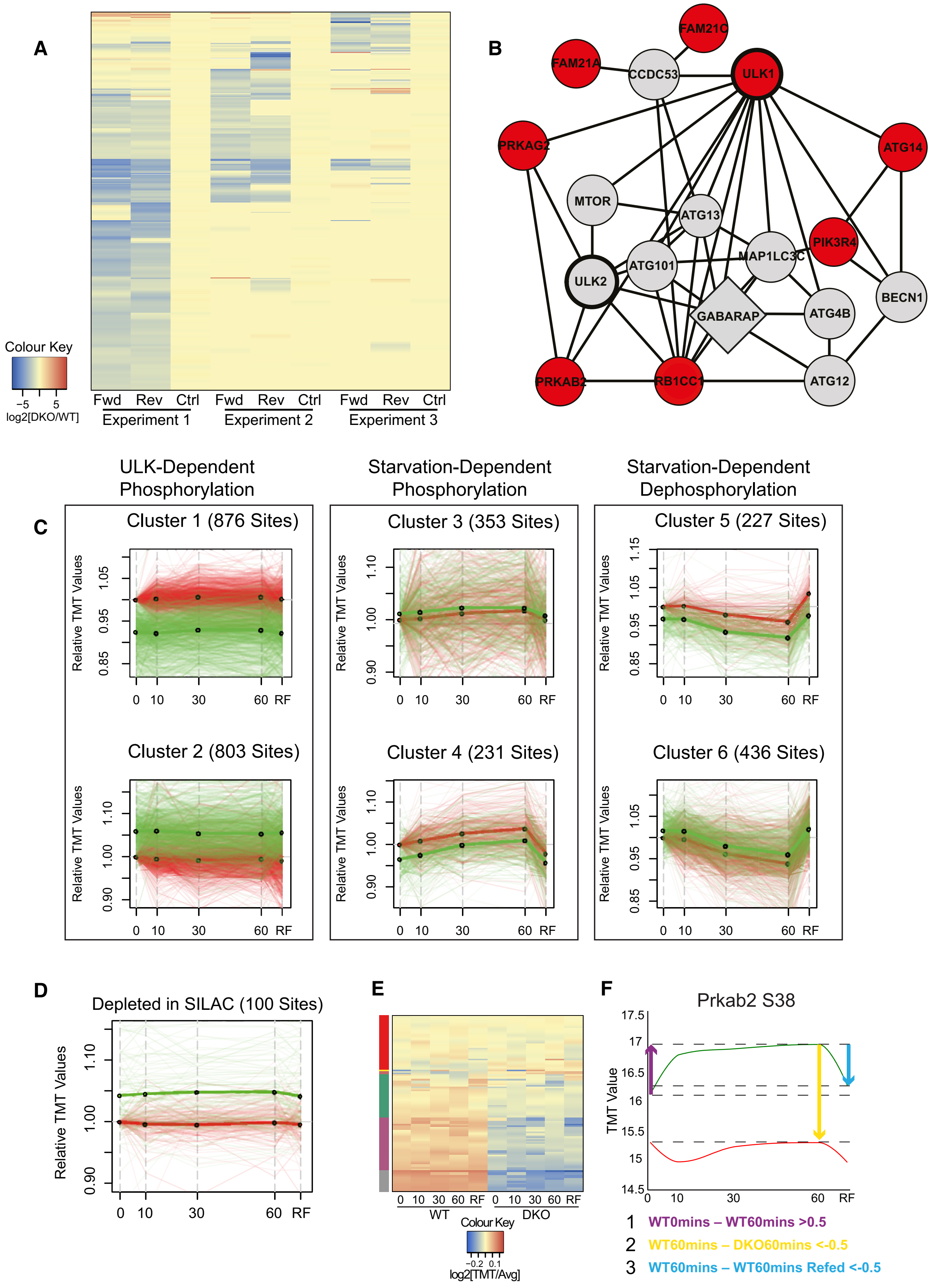
A
Colour Key
log2[DKO/WT]
Fwd Rev Ctrl
Experiment 1
Experiment 2
Experiment 3
B
FAM21C
FAM21A
CCDC53
ULK1
PRKAG2
ATG14
MTOR
ATG13
PIK3R4
ATG101
MAP1LC3C
ULK2
GABARAP
ATG4B
BECN1
PRKAB2
RB1CC1
ATG12
C
ULK-Dependent Phosphorylation
Starvation-Dependent Phosphorylation
Starvation-Dependent Dephosphorylation
Cluster 1 (876 Sites)
Cluster 2 (803 Sites)
Cluster 3 (353 Sites)
Cluster 4 (231 Sites)
Cluster 5 (227 Sites)
Cluster 6 (436 Sites)
Relative TMT Values
D
Depleted in SILAC (100 Sites)
E
WT
DKO
Colour Key
log2[TMT/Avg]
F
Prkab2 S38
TMT Value
1 WT0mins – WT60mins >0.5
2 WT60mins – DKO60mins <-0.5
3 WT60mins – WT60mins Refed <-0.5

Figure EV1.

Figure EV2. Peptide array-based *in vitro* kinase assays reveal direct ULK substrates.

A Controls for peptide array-based *in vitro* kinase assays to triage candidate ULK substrates are shown. Loading control (LC) and elution (Elu) samples for ULK complex samples used in assay were analysed by immunoblot.

B Autoradiograms of identical peptide arrays phosphorylated *in vitro* by the Ulk1 (top) or Ulk2 complex (bottom) in the presence or absence of an ULK inhibitor (MRT68921, 1 μM). Peptides used are listed in Appendix Table S2. Unannotated arrays are shown to demonstrate overall patterns of phosphorylation.

C Autoradiograms from B were sectioned and annotated. All peptides are shown with the same exposure.

© 2021 The Authors

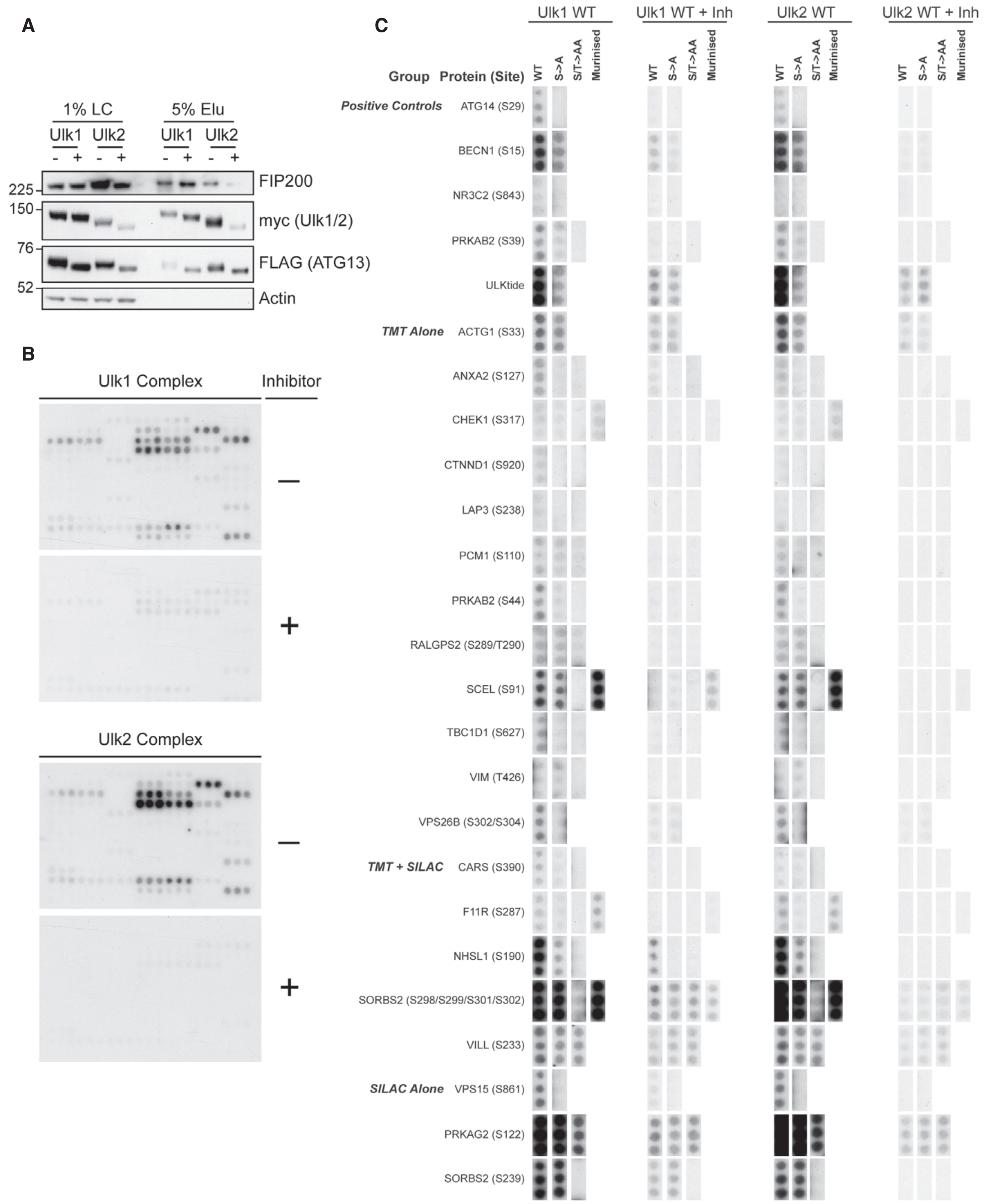
A
1% LC
5% Elu
Ulk1
Ulk2
Ulk1
Ulk2
- + - + - + - +
225
150
76
52
FIP200
myc (Ulk1/2)
FLAG (ATG13)
Actin
B
Ulk1 Complex
Inhibitor
Ulk2 Complex
–
+
–
+
C
Ulk1 WT
Ulk1 WT + Inh
Ulk2 WT
Ulk2 WT + Inh
WT
S->A
S/T->AA
Murinised
Group
Protein (Site)
Positive Controls
ATG14 (S29)
BECN1 (S15)
NR3C2 (S843)
PRKAB2 (S39)
ULKtide
TMT Alone
ACTG1 (S33)
ANXA2 (S127)
CHEK1 (S317)
CTNND1 (S920)
LAP3 (S238)
PCM1 (S110)
PRKAB2 (S44)
RALGPS2 (S289/T290)
SCEL (S91)
TBC1D1 (S627)
VIM (T426)
VPS26B (S302/S304)
TMT + SILAC
CARS (S390)
F11R (S287)
NHSL1 (S190)
SORBS2 (S298/S299/S301/S302)
VILL (S233)
SILAC Alone
VPS15 (S861)
PRKAG2 (S122)
SORBS2 (S239)

Figure EV2.

Figure EV3. CRISPR-based mutation of VPS15 ATP-binding site results in knockout phenotype.

A Control and VPS15 CRISPR cells were starved for 1 h before the localisation of WIPI2 and VPS15 (#NBP1-30463) were assessed with indicated antibodies. In VPS15 KO, the Golgi stain remains indicating the antibody is not specific for VPS15. Dashed boxes reveal magnified regions of interest.

B VPS15 KOs transfected with empty vector (EV) or VPS15-HA were fixed and LC3 (green), p62 (red) and HA (blue) were visualised. LC3 and p62 accumulation was reduced in VPS15-expressing cells.

C Pie charts reveal VPS15 (gene name *PIK3R4*) allele frequency in 6 VPS15 CRISPR clones. The key lists the absolute numbers detected for each allele when TOPO TA clones were sequenced, with total numbers of sequenced clones given in the centre. Allele phenotypes are annotated on the outside of each chart (f.s. = frameshift).

D ATP-binding site motif #PS00107 from ExPASy Prosite (Sigrist *et al*, 2013). ATP-binding sites from human VPS15 (amino acid positions 32–53) and *S. cerevisiae* Vps15p (amino acid positions 33–54) are given below with conforming amino acids in green, non-conforming amino acids in red and those for which any residue is acceptable in grey. Valine 50 and the corresponding yeast residue I51 are highlighted.

E HEK293A and VPS15 KO cells expressing VPS15-HA WT, ΔV50 or empty vector (EV) were cultured in full medium (F) or starved for 1 h with 100 nM Bafilomycin A1 (SB) before lysis and Western blot analysis. Only WT expression rescued VPS34 destabilisation (top), LC3 lipidation (bottom) and p62 accumulation.

Data information: Scale bars 10 µm.

 © 2021 The Authors

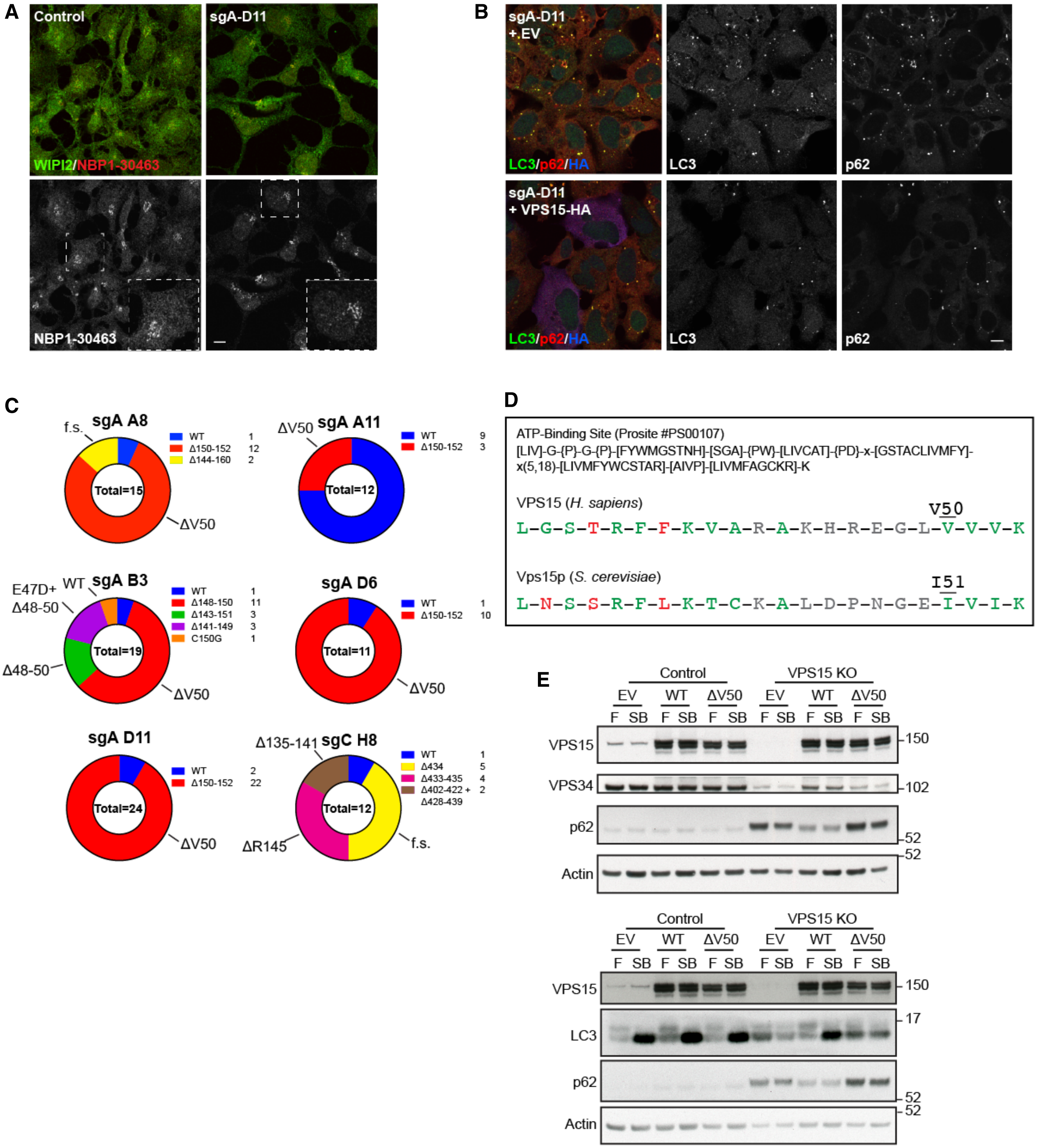
A
Control
sgA-D11
WIPI2/NBP1-30463
NBP1-30463
B
sgA-D11 +.EV
sgA-D11 + VPS15-HA
LC3/p62/HA
LC3
p62
C
sgA A8
f.s.
ΔV50
Total=15
WT 1
Δ150-152 12
Δ144-160 2
sgA A11
ΔV50
Total=12
WT 9
Δ150-152 3
sgA B3
E47D+ Δ48-50
WT
Δ48-50
ΔV50
Total=19
WT 1
Δ148-150 11
Δ143-151 3
Δ141-149 3
C150G 1
sgA D6
ΔV50
Total=11
WT 1
Δ150-152 10
sgA D11
ΔV50
Total=24
WT 2
Δ150-152 22
sgC H8
Δ135-141
ΔR145
f.s.
Total=12
WT 1
Δ434 5
Δ433-435 4
Δ402-422 + Δ428-439 2
D
ATP-Binding Site (Prosite #PS00107)
[LIV]-G-{P}-G-{P}-[FYWMGSTNH]-[SGA]-{PW}-[LIVCAT]-{PD}-x-[GSTACLIVMFY]-x(5,18)-[LIVMFYWCSTAR]-[AIVP]-[LIVMFAGCKR]-K
VPS15 (H. sapiens)
V50
L-G-S-T-R-F-F-K-V-A-R-A-K-H-R-E-G-L-V-V-V-K
Vps15p (S. cerevisiae)
I51
L-N-S-S-R-F-L-K-T-C-K-A-L-D-P-N-G-E-I-V-I-K
E
Control
VPS15 KO
EV
WT
ΔV50
F SB
VPS15
VPS34
p62
Actin
LC3
150
102
52
17

Figure EV3.

Figure EV4. Biochemical characterisation of VPS34 complexes reconstituted with VPS15 phosphomutants.

A VPS15 KO cells were transfected with VPS34, Beclin1, ATG14 and with either wild type (WT), 6SA or 6SE VPS15 and starved for 60 min as indicated before lysis and VPS34 complex coimmunoprecipitation via VPS15. Compared to WT, the *in vitro* lipid kinase activity of VPS34 complex I incorporating 6SA or 6SE VPS15 was reduced on ~800 nm large unilamellar vesicles (LUVs). PI3P was detected via a GST overlay assay using the GST-PX probe. Reaction mixtures were analysed by Western blot (right-hand side) to show VPS34 complex amounts used, a representative experiment of three independent repeats is shown. Dashed lines indicated where blots were cropped for presentation.

B HEK293A were cotransfected with VPS34, Beclin1, ATG14, UVRAG and with either empty vector (EV) or wild type (WT), 6SA or 6SE VPS15. After starvation for 30 min, cells were lysed and relative VPS34 complex component immunopurification via VPS15 was assessed via immunoblotting. Quantifications represent mean ± SEM, $n = 3$.

C No difference in *in vitro* lipid kinase activity on 100 nm LUVs was detected with VPS34 complex I incorporating either VPS15 WT, 6SA or 6SE. ADP-Glo kinase assay (Promega V6930) was used to assess lipid kinase activity and mean ± SD from 4 technical repeats are shown. Data reflect representative data from 1 of 3 independent repeats.

D The membrane binding affinity of VPS34 CI incorporating either WT, 6SA or 6SA VPS15 on 100 nm LUVs (large unilamellar vesicles) was assessed via liposome floatation assay. Complexes segregating to the top (liposome-bound) and bottom (liposome-unbound) of a sucrose gradient were separated by SDS–PAGE and Coomassie-stained gels are shown. When the percentage of VPS15 and VPS34 in the top fraction was quantified, none of the differences between WT, 6SA or 6SE were significant. In = input; mean ± SD, $n = 3$.

Data information: (B) $*P < 0.05$, n.s. not significant, [one-tailed ANOVA]. (C, D) n.s. not significant, [Student's *t*-test].

 © 2021 The Authors

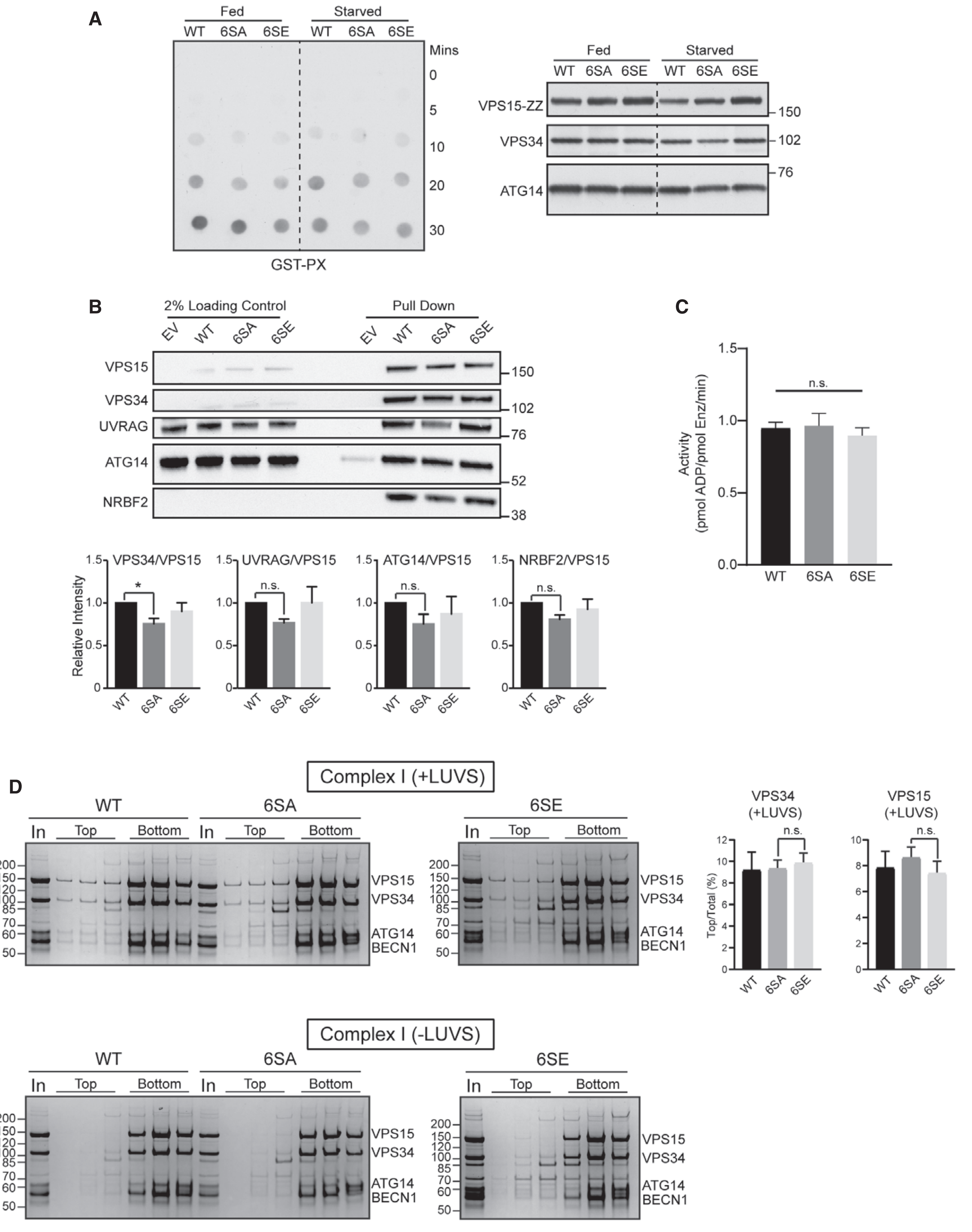

A
Fed
Starved
WT 6SA 6SE WT 6SA 6SE
Mins
0
5
10
20
30
GST-PX
VPS15-ZZ
VPS34
ATG14
150
102
76
B
2% Loading Control
Pull Down
EV WT 6SA 6SE EV WT 6SA 6SE
VPS15
VPS34
UVRAG
ATG14
NRBF2
150
102
76
52
38
VPS34/VPS15
UVRAG/VPS15
ATG14/VPS15
NRBF2/VPS15
Relative Intensity
n.s.
*
C
Activity (pmol ADP/pmol Enz/min)
n.s.
WT 6SA 6SE
D
Complex I (+LUVS)
WT
6SA
6SE
In Top Bottom
VPS15
VPS34
ATG14
BECN1
VPS34 (+LUVS)
VPS15 (+LUVS)
Top/Total (%)
Complex I (-LUVS)

Figure EV4.

Figure EV5. VPS15 knockout clones reveal VPS15 S861-dependent phenotypes.

A Wild type (WT), 6SA, 6SE, 5SA or 5SE VPS15-HA, or empty vector (HA) were expressed in VPS15 KO cells as indicated. Cells were starved for 1 h, and the distributions of WIPI2 (green) and HA (red) were assessed. Quantification of WIPI2 puncta number per cell, mean ± SEM, n = 2, 3,483–5,297 cells quantified per condition. Representative Western blots are shown.

B Stably rescued VPS15 KO (sgA-D6), HEK293A and VPS15 KO control cells were starved for 1 h with (SB) or without (St) 100 nM Bafilomycin A1, or cultured in full medium (F). Quantification shows LC3II/Actin, mean ± SEM, n = 5.

C HEK293A or VPS15 KO cells were treated with full medium (F), EBSS (St, 2 h) or Deferiprone (DFP, 1 mM, 6 h) in the presence or absence of MRT68921 (1 μM).

D HEK293A were starved for 1 h (St), treated with Oligomycin and Antimycin (O/A, 1 μM) or DFP (1 mM) for 6 or 24 h, or cultured in full medium (F) as specified before lysis and Western blot analysis.

E HEK293A were transfected with RISC-free (RF) or *siPIK3R4* (si) before treatment with VPS34-IN1 (IN1, 1 μM) or EBSS (Starved) for 2 h. To show VPS34 levels, identical samples were loaded in parallel on separate gels, indicated by dashed line. M = protein marker.

Data information: (A, B) *P < 0.05, **P < 0.01, ***P < 0.001, n.s. not significant, [one-tailed ANOVA]. (A) Scale bar 10 μm.

 © 2021 The Authors

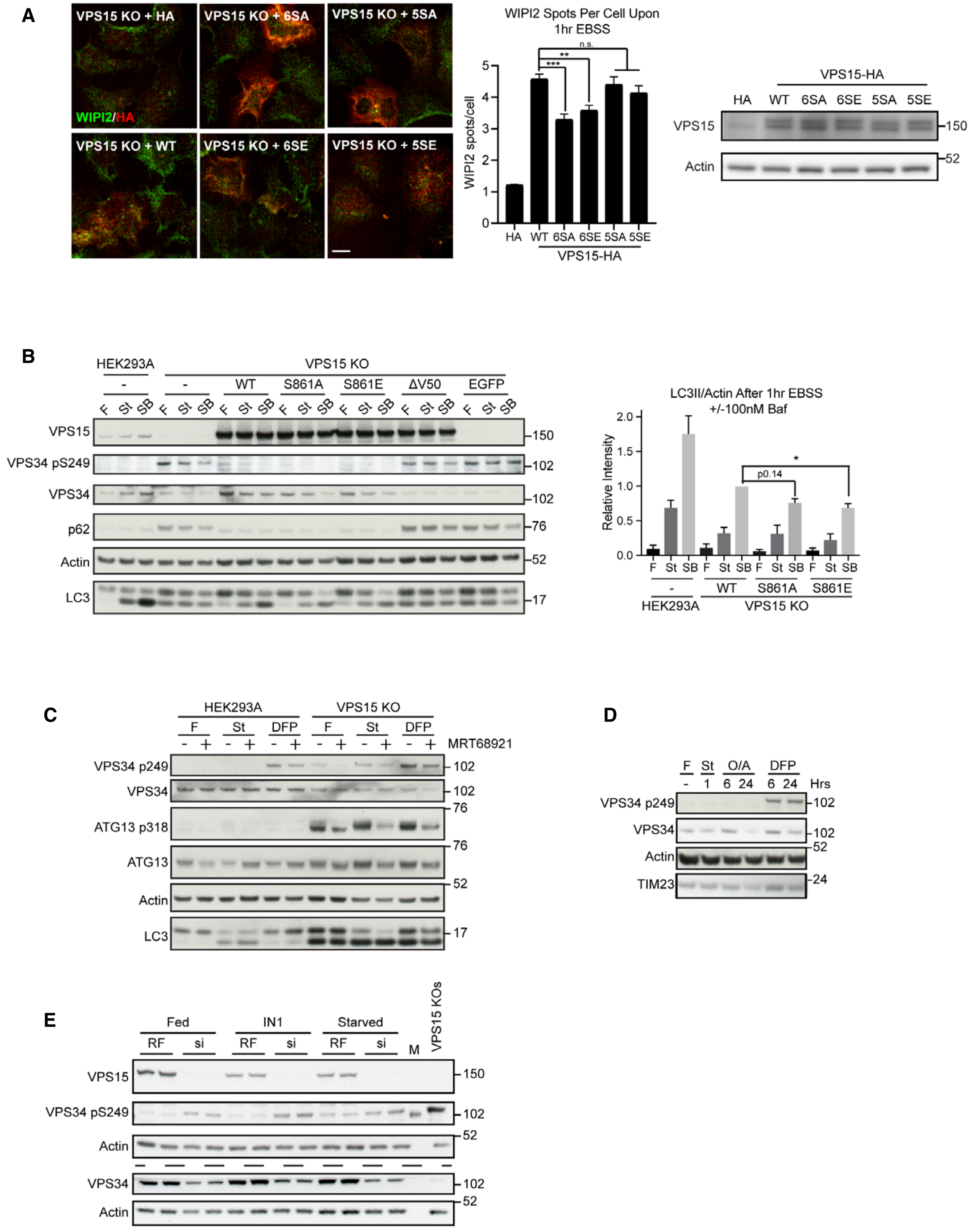
A
VPS15 KO + HA
VPS15 KO + 6SA
VPS15 KO + 5SA
WIPI2/HA
VPS15 KO + WT
VPS15 KO + 6SE
VPS15 KO + 5SE
WIPI2 Spots Per Cell Upon 1hr EBSS
n.s.
**
***
WIPI2 spots/cell
0
1
2
3
4
5
HA WT 6SA 6SE 5SA 5SE
VPS15-HA
VPS15-HA
HA WT 6SA 6SE 5SA 5SE
VPS15
Actin
150
52
B
HEK293A
VPS15 KO
- - WT S861A S861E ΔV50 EGFP
F St SB
VPS15
VPS34 pS249
VPS34
p62
Actin
LC3
150
102
102
76
52
17
LC3II/Actin After 1hr EBSS +/-100nM Baf
Relative Intensity
0.0
0.5
1.0
1.5
2.0
p0.14
*
F St SB
- WT S861A S861E
HEK293A VPS15 KO
C
HEK293A VPS15 KO
F St DFP F St DFP
- + MRT68921
VPS34 p249
VPS34
ATG13 p318
ATG13
Actin
LC3
102
102
76
76
52
17
D
F St O/A DFP
- 1 6 24 6 24 Hrs
VPS34 p249
VPS34
Actin
TIM23
102
102
52
24
E
Fed IN1 Starved
RF si RF si RF si M
VPS15 KOs
VPS15
VPS34 pS249
Actin
VPS34
Actin
150
102
52
102
52

Figure EV5.
