# Supplementary figures and images for: Phosphoproteomic identification of ULK substrates reveals VPS15‐dependent ULK/VPS34 interplay in the regulation of autophagy
Source: EMBO J. 2021 Jun 14;40(14):e105985. doi: 10.15252/embj.2020105985 (PMC8280838; doi:10.15252/embj.2020105985)

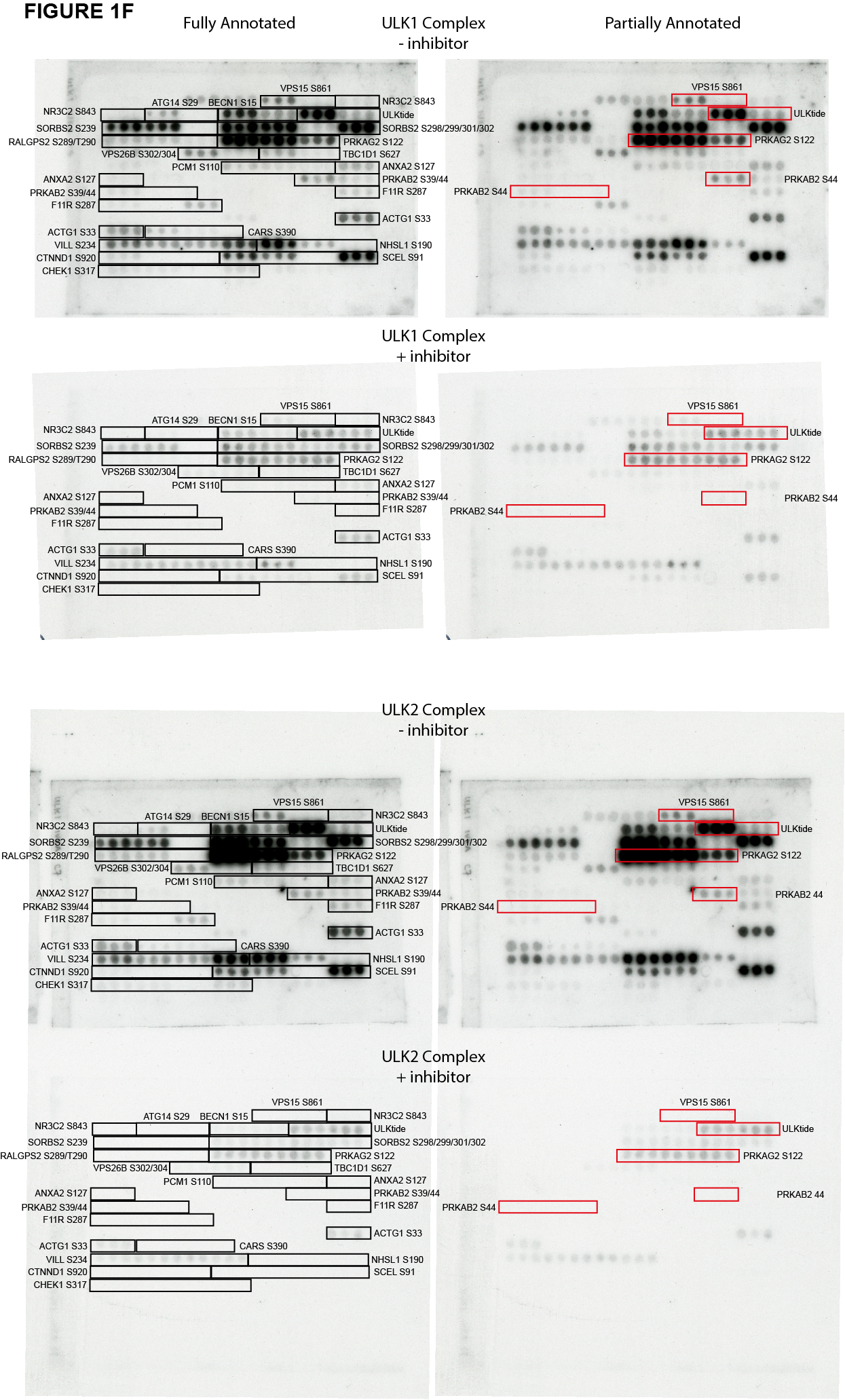

Supplement: Supplementary file 5 — Source Data for Figure 1 [file EMBJ-40-e105985-s002.tif]

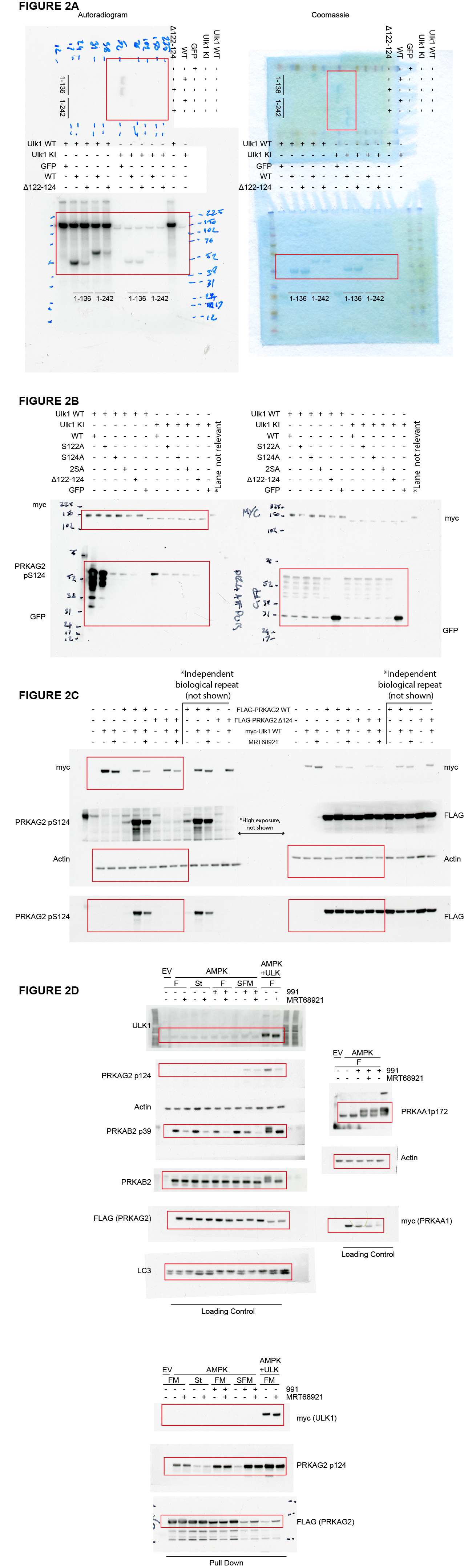

Supplement: Supplementary file 6 — Source Data for Figure 2 [file EMBJ-40-e105985-s006.tif]

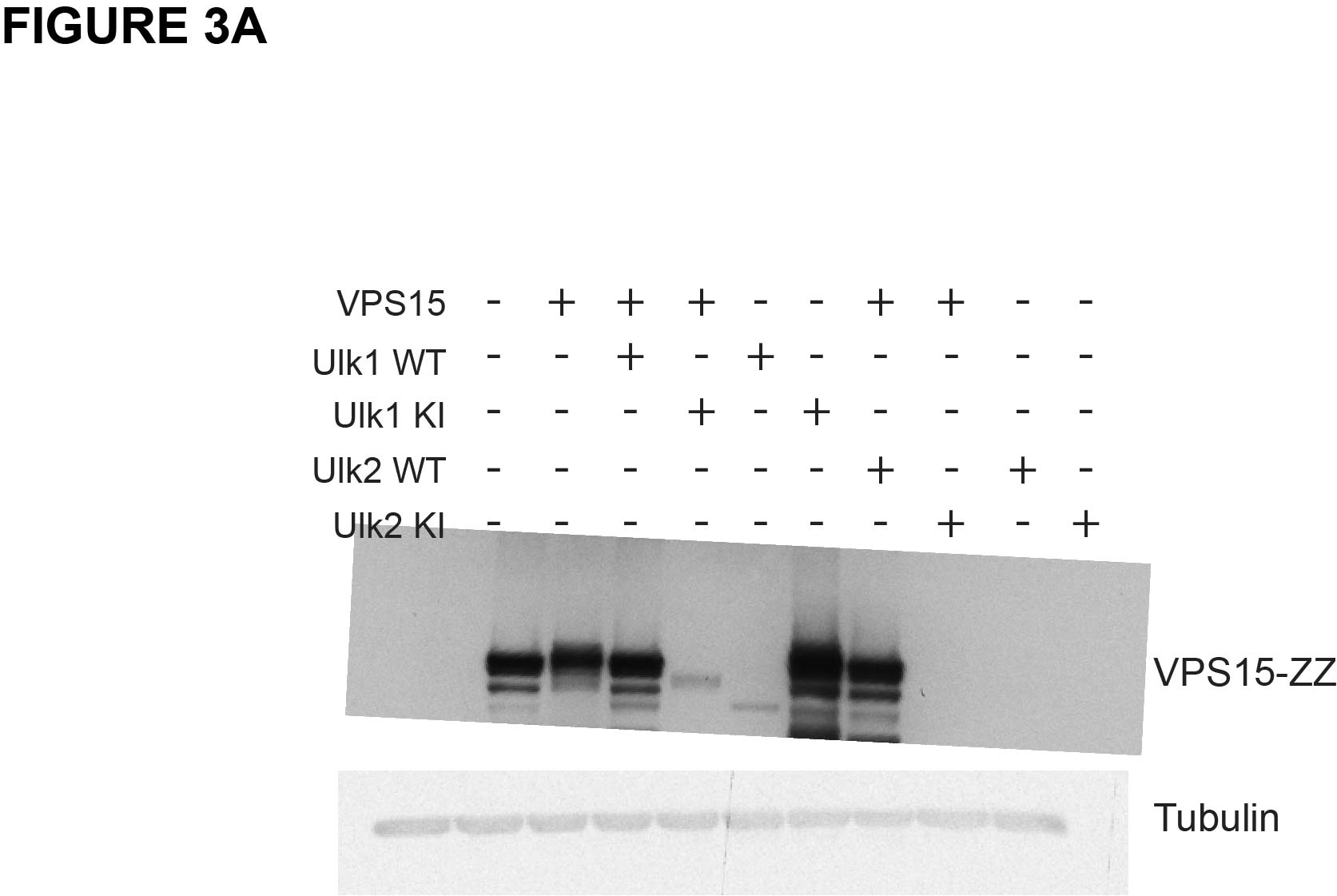

Supplement: Supplementary file 7 — Source Data for Figure 3 [file EMBJ-40-e105985-s010.jpg]

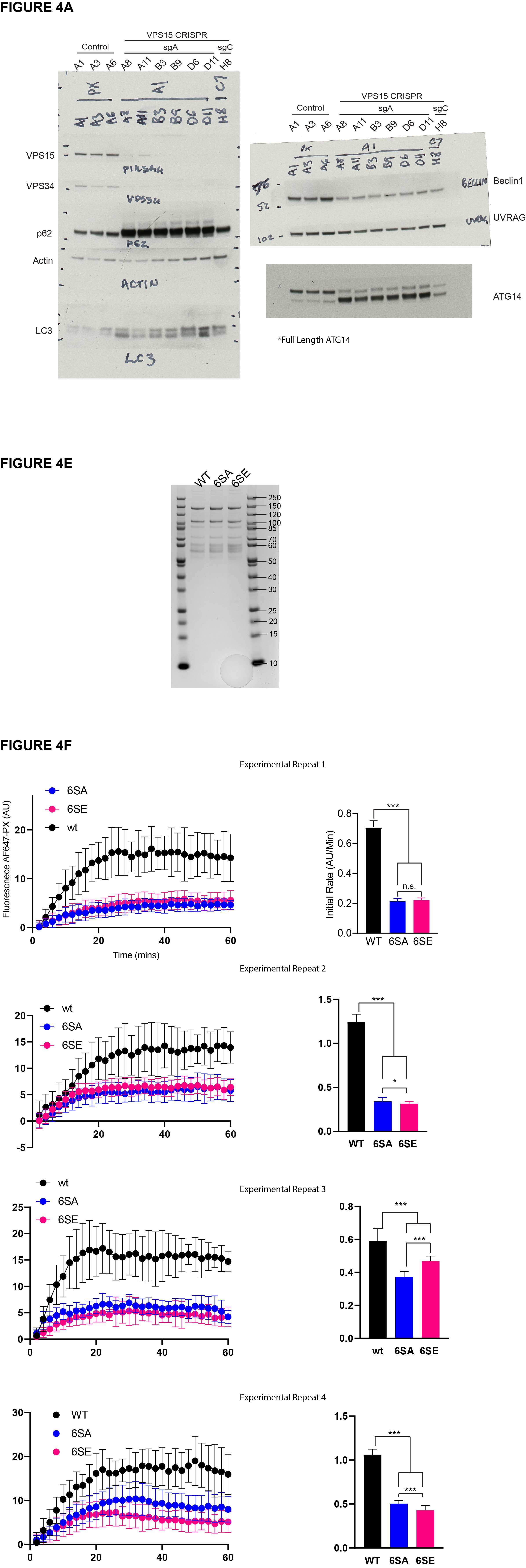

Supplement: Supplementary file 8 — Source Data for Figure 4 [file EMBJ-40-e105985-s001.jpg]

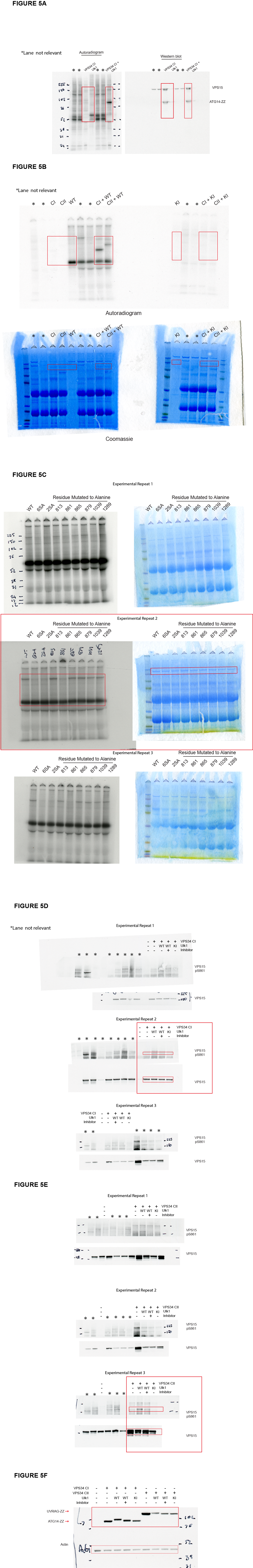

Supplement: Supplementary file 9 — Source Data for Figure 5 [file EMBJ-40-e105985-s005.tif]

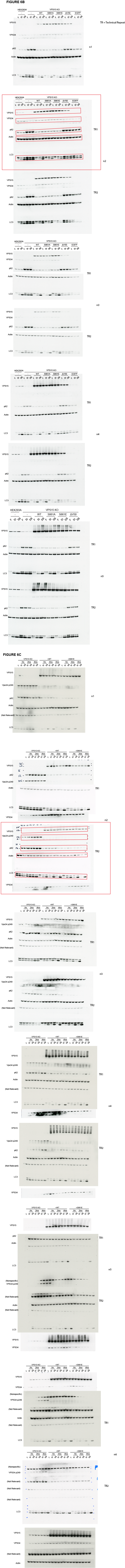

Supplement: Supplementary file 10 — Source Data for Figure 6 [file EMBJ-40-e105985-s011.tif]

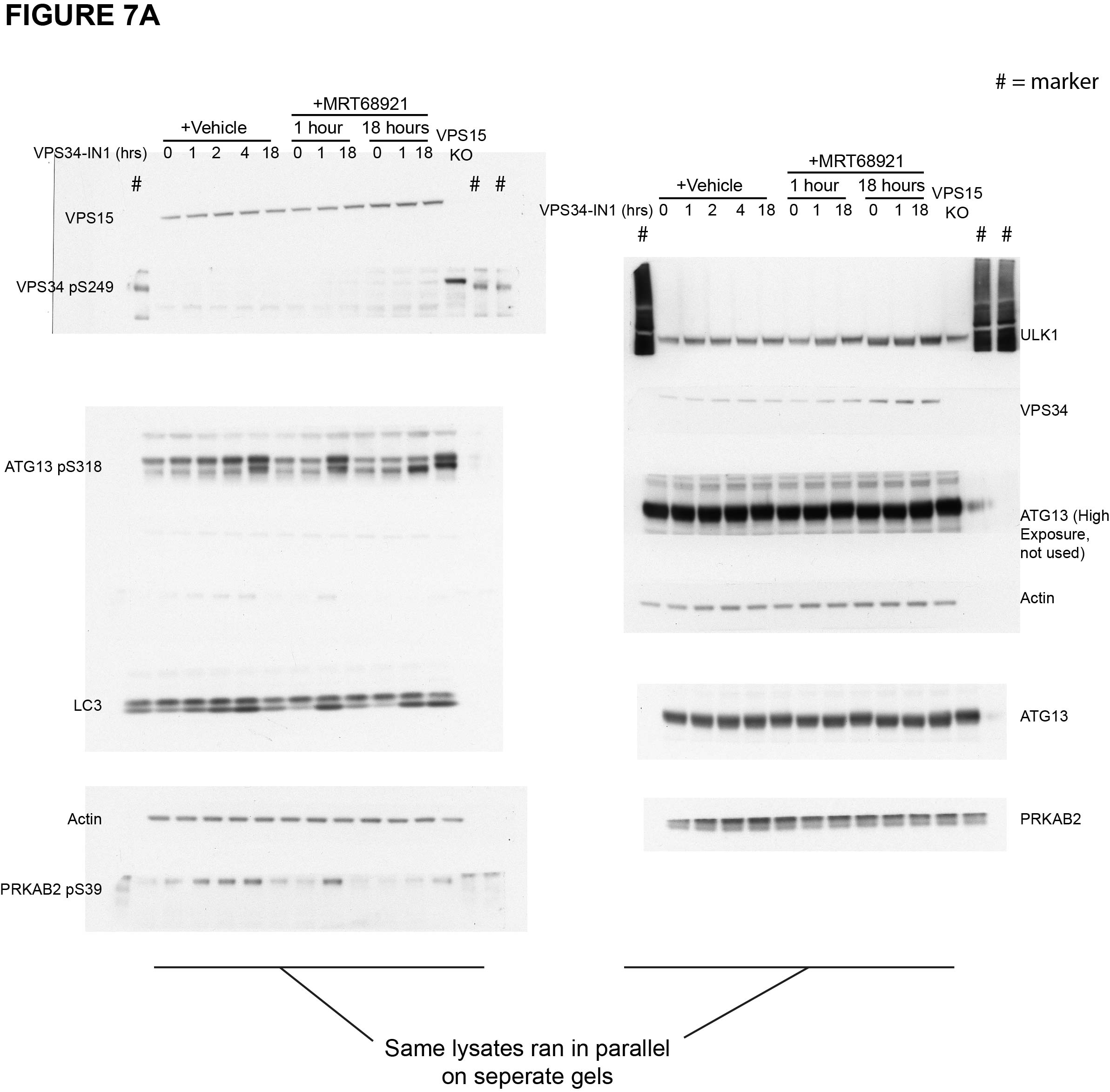

Supplement: Supplementary file 11 — Source Data for Figure 7 [file EMBJ-40-e105985-s004.jpg]
